# Polyamines in Dysbiotic Oral Conditions of Older Adults: A Scoping Review

**DOI:** 10.3390/ijms251910596

**Published:** 2024-10-01

**Authors:** Stephanie Chu, Alice Kit Ying Chan, Chun Hung Chu

**Affiliations:** Faculty of Dentistry, The University of Hong Kong, Hong Kong 999077, China

**Keywords:** older adults, elderly, oral health, oral cancer, periodontal, halitosis, prevention, polyamines, biomarkers

## Abstract

Polyamines modulate cellular proliferation and function. Their dysregulation results in inflammatory and oncological repercussions. This study aims to map the current literature and provide an overview of polyamines in dysbiotic oral conditions among older adults. English publications indexed in MEDLINE, Scopus, and Web of Science from January 2000 to May 2024 were screened. Eligibility criteria included clinical and laboratory studies using samples from adults aged 65 or above. This scoping review identified 2725 publications and included 19 publications. Ten studies detected that older adults with oral carcinoma had increased levels of polyamines such as spermidine in saliva and tumour-affected tissues. Eight studies reported older adults suffering from periodontal infection had increased levels of polyamines such as putrescine in saliva, gingival crevicular fluid, and biofilm from the gingival crevice. Two studies showed polyamine levels could reflect the success of periodontal therapy. Three studies found older adults with halitosis had increased levels of polyamines such as cadaverine in saliva and tongue biofilm. Polyamines were suggested as biomarkers for these oral conditions. In conclusion, certain polyamine levels are elevated in older adults with oral cancer, periodontal infections, and halitosis. Polyamines may be used as a simple and non-invasive tool to detect dysbiotic oral conditions and monitor treatment progress in older adults (Open Science Framework registration).

## 1. Introduction

Ageing is among the plethora of physiological processes influenced by polyamine activity. Age-associated reductions of polyamine levels contribute to physical and cognitive decline [1]. For example, spermine degradation by spermine oxidase increases with age and is associated with cellular senescence [1]. The Global Burden of Disease, Injuries, and Risk Factors study in 2017 reported that 31% of all diseases were age-associated, mainly of cardiovascular, neurodegenerative, musculoskeletal, arthritis, and cancer origin [2].

Dysbiotic oral conditions correlate with systemic disorders among older adults [3]. For example, comorbidities of periodontitis include age-associated disorders such as diabetes. Having a bi-directional relationship, diabetes increases the incidence and progression of periodontitis [4]. Periodontitis is a prevalent oral disease causing pain, infection, and eventual tooth loss. Tooth loss is associated with the risks of malnutrition, depression, and dementia [5]. Oral health is deemed an integral counterpart of healthy ageing, and dysbiotic oral conditions significantly hinder the well-being and quality of life of older adults [6].

Polyamines are organic compounds with a molecular construct of amino groups positioned at both ends of a hydrocarbon [7,8]. They can be diamines such as putrescine and cadaverine, tri-amines such as spermidine, and tetra-amines such as spermine. Natural polyamines are synthesized by human cells and intestinal microbiota [9]. They modulate cell proliferation, differentiation, growth, and function [10,11,12]. Their ubiquitous influence on cellular activity cascades into diverse physiological systems. 

Polyamines support cardiovascular, neurological, gastrointestinal, renal, endocrine, and cognitive performance in mammalian systems and thus are pertinent to the overall health and survival of the organism [13]. Their dysregulation results in inflammatory, pathogenic, and oncological repercussions.

While the diagnostic and therapeutic potential of polyamines have been extensively researched for systemic health, their mechanisms in the context of oral health remain unclear, particularly in dysbiotic oral conditions. The oral cavity houses a complex reservoir of microbes which co-exist with host cells in equilibrium under healthy conditions. However, dysbiosis can be triggered by microbial, inflammatory, or autoimmune factors that result in an array of oral diseases [14,15,16]. The presence, levels, and functions of intra-oral polyamines are critical to further understand the nature of oral conditions commonly found among older adults. The objective of this study is to map the current literature and provide an overview of polyamines in dysbiotic oral conditions among older adults.

## 2. Methods

This review was reported according to the Preferred Reporting Items for Systematic Review and Meta-analysis (PRISMA) Extension for Scoping Reviews (PRISMA-ScR) checklist. The review protocol was registered with Open Science Framework (Registration DOI: https://doi.org/10.17605/OSF.IO/67UNB). The research question for this study was: “What are the associations between dysbiotic oral conditions of older adults and polyamines in the current literature?”.

### 2.1. Search Strategy

Two researchers (SC and AKC) independently searched three electronic databases (MEDLINE, Scopus, and Web of Science) for English publications on or after 1 January 2000 using the keywords (polyamine OR putrescine OR spermidine OR spermine OR cadaverine) AND (oral disease OR oral health OR oral biomarkers OR oral metabolites OR oral microorganisms OR oral biofilm OR periodontal OR periodontal disease OR oral tumour OR oral cancer OR dental caries). The last literature search was completed on 28 May 2024. The full search strategy is presented in the Supplementary file. An additional literature search was conducted on the reference lists of the included publications.

### 2.2. Study Selection

After removing the duplicates, two researchers screened the titles and abstracts of the retrieved publications independently according to the inclusion and exclusion criteria listed in Table 1. Full texts of the identified publications or those whose eligibility could not be decided from titles and abstracts were assessed for suitability. The third researcher (CHC) was consulted in the case of a disagreement during the selection process.

### 2.3. Data Extraction and Synthesis

Two researchers (SC and AKC) collectively determined the variables deemed necessary for data extraction. Data were retrieved independently by the two researchers recorded on a pre-defined spreadsheet for data collection. The first author’s name, year of publication, country, type and methodology of study, sample source, dysbiotic oral condition investigated, and potential application of each publication were collected. The main findings of each publication were reviewed sequentially and stratified by dysbiotic oral condition. Outcomes included types of polyamines or related molecules identified and the changes in their levels in dysbiotic oral conditions.

## 3. Results

**Figure 1:**
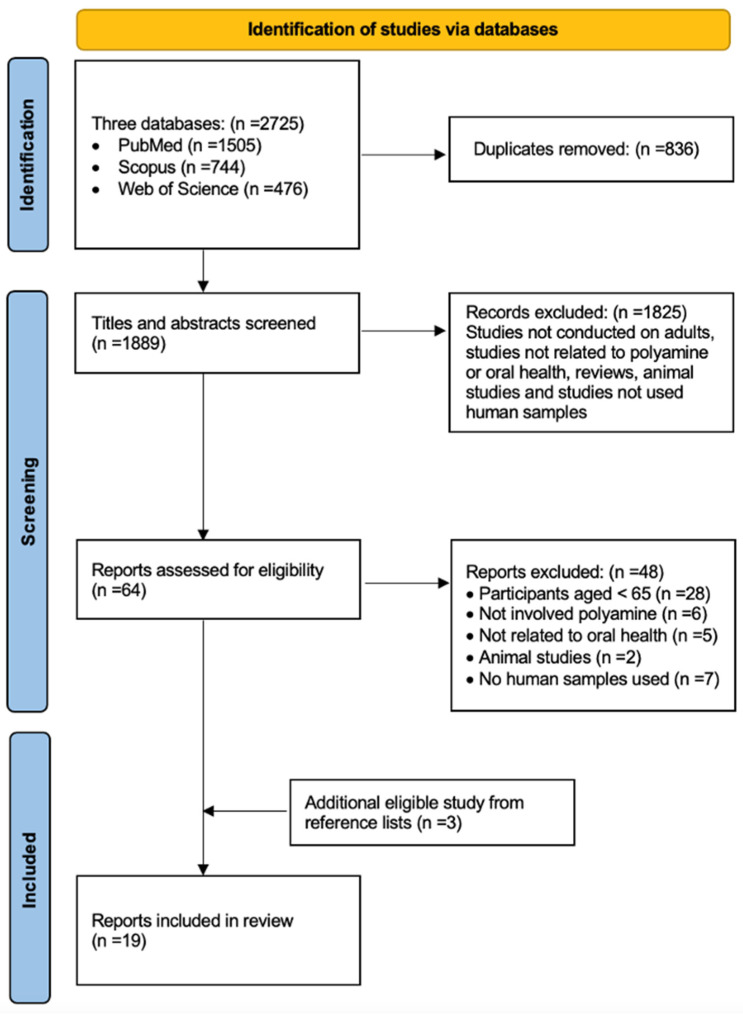
Shows the flowchart of the literature search.

A total of 2725 articles were retrieved from three electronic databases and 19 publications included in this review (Figure 1). The two researchers performed searches and extracted data independently with 97% agreement. The included studies were conducted in North and South America (n = 7), Asia (n = 8), and Europe (n = 4). Table 2 summarises the changes identified in the levels of polyamines and related molecules in dysbiotic oral conditions of older adults.

Table 3 shows the sources of dysregulated polyamines and related molecules in dysbiotic oral conditions of older adults. All studies involved the collection of biological fluids (saliva or gingival crevicular fluid), tissue, or biofilm from the oral cavity of older adults for in vitro metabolomic or transcriptomic analyses. Only one study incorporated in vivo analysis by means of a chairside colorimetric test to assess the salivary levels of polyamines. The studies investigated oral cancer (n = 10), periodontal infection (n = 8), and halitosis (n = 3). All studies reported the dysregulation of polyamines. They investigated the potential application of polyamines in the detection of dysbiotic oral conditions, with two further exploring its use in monitoring treatment outcomes.

**Figure 2:**
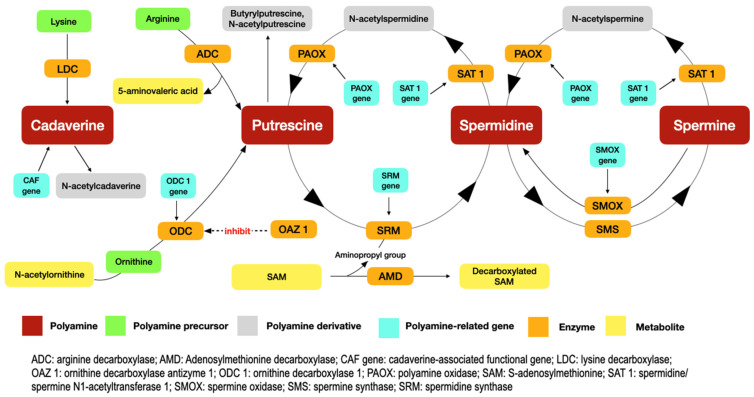
The constituents of the polyamine pathway reported in the included studies.

Figure 2 summarises constituents of the polyamine pathway reported in the 19 studies included in this review. Polyamines are formed from the decarboxylation of amino acids. Putrescine, the precursor of spermidine and spermine, is formed from either the decarboxylation of ornithine by ornithine decarboxylase (ODC) or arginine by arginine decarboxylase (ADC). Cadaverine is produced from the decarboxylation of lysine by lysine decarboxylase (LDC). A number of enzymes are produced from their corresponding genes to catalyse the production and conversion of polyamines. For example, the ODC1 gene produces ODC. Apart from polyamines and their related genes and enzymes, researchers also examined the levels of their derivatives and metabolites as biomarkers for oral cancer, periodontal infection, and halitosis.

### 3.1. Oral Cancer

World Cancer Research Fund International reported oral cancer to have a worldwide incidence of over 389,000 in 2022 and a mortality rate of close to 50% [35,36]. Oral squamous cell carcinoma (OSCC) is the most common histological type accounting for over 90% of all cases. Survival rates of 80–90% have been reported among patients diagnosed at the first stage of OSCC, as opposed to 20–30% rates at the third or fourth stages [25,37]. The lack of symptoms at early stages often results in late diagnosis and the subsequent compromise in prognosis and treatment outcomes. Furthermore, older age has a substantial influence on survival and is a significant predictor of worse prognosis at all stages compared to younger adults [37].

The World Health Organization has stressed that early detection of oral cancer is critical to reduce its adverse sequalae and improve the success of therapeutic management [18,23]. Thus, researchers are examining the use of polyamines to identify oral cancer in its early stages [20,23,38]. Nine out of ten in vitro studies utilized saliva samples for metabolomic analysis. The non-invasive approach, ease, accessibility, and cost-effectiveness of obtaining saliva samples enable its use as an alternative diagnostic tool for OSCC [20]. Furthermore, salivary assessment can be useful for oral cancer detection because saliva is in direct contact with oral lesions [38]. However, external factors such as medications, dietary intake, or circadian rhythm may modify the assessment outcomes.

Polyamines, their related genes, and metabolites increase in oral cancer. Their upregulation supports the cellular proliferation necessary for tumour growth and enhances its malignant potential [39]. Song et al. found that putrescine and cadaverine were among the top metabolites to undergo significant upregulation in malignant transformation [17]. This is partially attributed to dysregulation of their precursors, arginine and lysine, respectively. Spermidine, N-acetylputrescine, N-acetylcadaverine, N-acetylspermidine and N1-acetylspermine were also elevated in other included studies [17,21,22,23,24,25] (Figure 2).

Hsu et al. compared the profiles of paired cancerous and adjacent non-cancerous tissues from OSCC patients to perform thorough metabolomic and transcriptomic analyses of the perturbed polyamine pathway [23]. They found a significant increase in the expression of polyamine-related genes ODC1, SRM, SMS, SAT1, and spermine oxidase in tumour-affected tissues. ODC1 serves as a key rate-limiting enzyme of polyamine biosynthesis and directly influences putrescine production. Although they reported elevated levels of ornithine in cancer-affected tissue, Alves et al. and Song et al. noted its reduction in saliva during malignant changes [17,25]. This reduction of ornithine may be ascribed to its increased conversion to putrescine from the upregulation of gene ODC1.

The elevation of putrescine levels was also found to correlate with the unaltered expression of gene AMD1 [23]. Its product, AMD1, is a rate-limiting enzyme of spermidine and spermine by catalysing the decarboxylation of S-adenosylmethionine (SAM), which then donates the aminopropyl group necessary for the conversion of putrescine to spermidine. The aminopropyl group is conjugated with putrescine via spermidine synthase (SRM) to form spermidine, which is then further converted to spermine by spermine synthase (SMS). The elevation of spermidine production was slight relative to that of putrescine in the study as it was likely limited by unaltered AMD1 levels. This is consistent with findings by Ishikawa et al., who also reported a slight increase of spermidine compared to that of putrescine in OSCC tissues [21].

Upregulation of the polyamine pathway is also observed in malignant changes of precancerous lesions. In the malignant change of oral leukoplakia with dysplasia, Michailidou et al. revealed an increase in salivary mRNAs encoding ornithine decarboxylase antizyme 1 (OAZ1) and spermidine/spermine N1-acetyltransferase 1 (SAT1). OAZ1 and SAT1 are key regulators of polyamine synthesis [18]. OAZ1 inhibits ODC and the uptake of polyamines. It is critical for DNA repair and the regulation of cell growth. OAZ1 also affects the metastatic potential of the human OSCC cell line [26]. SAT1 facilitates the catabolism of spermine to spermidine for cellular efflux or the conversion back to putrescine [40]. Several studies have suggested that the expression of enzymes OAZ1 and SAT1 is instigated by intracellular polyamine activity [38,41,42,43]. Thus, this enzymatic upregulation in malignant change reflects an associated increase in polyamine levels. Researchers used salivary OAZ1 and SAT1 as biomarkers for the detection of OSCC in early stages. A predictive ability of 80% was achieved when these biomarkers were analysed together with IL-8 and IL-1B mRNA [18]. However, Cheng et al. observed that the increase of OAZ1 and SAT1 in OSCC patients was not significantly different to that found in smokers with periodontitis. This generates reservations for the reliability of OAZ1 and SAT1 as biomarkers for OSCC in patients with concomitant periodontitis and smoking habits [26].

Two included studies also evaluated polyamine dysregulation in the malignant transformation of oral lichen planus [19,20]. Polyamine content and homeostasis influence the degree of inflammation in such lesions. Increased levels of OAZ1, putrescine, N-acetylputrescine and N1-acetylspermine were reported and may serve to detect malignant changes in lichenoid lesions. In addition, Cheng et al. also recognized that a history of OSCC carries risk for recurrence [20]. A significant increase of OAZ1 was reported in patients with newly detected OSCC compared to those in remission for at least two years. As the increase in polyamine activity upregulates OAZ1, this finding signifies the elevation of polyamines in malignant changes. Measurements of salivary OAZ1 levels may predict malignancy in patients with a past history of OSCC before symptoms arise, allowing earlier diagnosis, improved prognosis, and enhanced therapeutic outcomes.

### 3.2. Periodontal Infection

Periodontal infection is characterized by destruction of the supporting tissues of teeth. This is attributed to the dysbiotic relationship between the host immunity and the oral microbiota [44]. The World Health Organization (2021) deemed periodontal diseases as a significant oral health burden globally by causing pain, infection, and eventual tooth loss. Periodontal disease is the leading cause of tooth loss and a risk factor of mortality, in which progression is age-related [44]. A recent review showed 60% of those aged 65 and above suffered from periodontal disease [5]. Older adults are at increased risk due to cellular senescence and the associated decline in immune function. The high prevalence of periodontal disease among older adults denotes an expected rise in oral disease burden among the ageing population [45]. Polyamines are involved in inflammatory processes, cellular proliferation, tissue regeneration, and bone formation, all of which are affected in periodontal disease [46]. Thus, the perturbation of polyamines is one of the hallmarks of periodontal disease. Among the seven studies that investigated polyamine dysregulation in periodontal disease, three were conducted from gingival crevicular fluid samples, three from saliva, and one from biofilm collected at the gingival crevice. Gingival crevicular fluid has been considered the most proximal biofluid to reflect periodontal disease pathology, with its metabolites being accurate reporters of the pathophysiological state [47].

Subgingival microorganisms with proteolytic metabolism contribute to increased intraoral polyamines. Bacteria such as Fusobacterium, Prevotella, and Porphyromonas proteolyze host amino acids, including ornithine and lysine, to produce putrescine, cadaverine, spermine, and spermidine metabolites. These bacterially derived polyamines enhance their metabolism, communication, virulence, and resistance to antibiotics [48]. Andörfer et al. reported the association of plaque biofilm with upregulated levels of numerous polyamines including cadaverine, N-acetyl-cadaverine, butyrylputrescine, N-acetylputrescine, and diacetylspermidine [29]. Putrescine and cadaverine are both produced from the putrefactive processes of periodontal pathogens and associated with tissue decay. Barnes et al. revealed their elevated levels in diseased sites affected by gingivitis and periodontitis [49]. Rodrigues et al. found that putrescine levels represent the intensity of periodontal breakdown and degree of disease progression [27]. Ozeki et al. reported elevated concentrations of putrescine in deep periodontal pockets [28]. The derivative butyrylputrescine was associated with the greatest number of disease traits investigated by Andörfer et al., including probing pocket depths, clinical attachment levels, plaque, calculus, and halitosis. Notably, the upregulation of salivary butyrylputrescine was strongly associated with a five-year tooth loss [29].

While putrescine is a marked disease biomarker among older adults, other studies revealed that ornithine, spermidine, N-acetylspermidine, and cadaverine are significant in younger adults affected by periodontal disease. Such variations in the salivary metabolomic profiles may be attributed to differences in host responses and characteristics of chronic periodontal disease progression. This warrants further studies specific to older aged groups [27,46]. Bacterially derived cadaverine impairs intrinsic immunity and is upregulated in poor oral hygiene conditions. The subgingival niche shifts to favour gram-negative microbiota upon disease progression. It generates LDC, which affects host lysine metabolism to produce cadaverine and carbon dioxide. Cadaverine and its derivative N-acetyl-cadaverine were positively correlated to the signs and symptoms of halitosis, gingival inflammation, clinical attachment loss, increased pocket depth, and tooth loss in the included studies [29,31]. The depletion of host lysine by bacterial LDC impairs cellular turnover of the junctional epithelium, resulting in a damaged barrier permeable to bacterial invasion [46].

Andörfer et al. reported that salivary butyrylputrescine and cadaverine were increased in the presence of systemic inflammatory markers, which are often upregulated by periodontitis and its comorbidities [29]. Smoking was also significantly associated with increased salivary butyrylputrescine and cadaverine, which enhance bacterial virulence and periodontal destruction [31]. Smoking also increased levels of OAZ1 and SAT1 in periodontitis [26]. While periodontal inflammation often upregulates polyamine metabolites, Cheng et al. found remarkably low levels of salivary mRNAs encoding OAZ1 and SAT1 among non-smokers with periodontitis. As OAZ1 serves to inhibit ODC, this may provide explanation for the increased ODC levels reported in periodontitis [26]. The significantly low levels of OAZ1 and SAT1 reflect a unique trend of polyamine dysregulation in periodontal disease. This highlights their potential as salivary biomarkers in non-smoking, periodontitis-affected patients.

Two studies have shown that polyamine levels can reflect the success of periodontal therapy to monitor treatment progress [31,49]. Barnes et al. reported significant reductions of putrescine in gingival crevicular fluid after the use of a triclosan-containing dentifrice with known clinical efficacy against gingivitis [49]. This is supported by early studies, which reported significantly reduced levels of putrescine after effective periodontal treatments [50]. Levine et al. conducted a study among subjects suffering from chronic periodontitis which revealed increased cadaverine fractions among poor responders of non-surgical periodontal therapy [31]. Attachment loss is associated with a depletion of biofilm lysine and an increase in cadaverine from bacterial LDC activity. Thus, it was suggested that measurements of biofilm lysine and cadaverine content can reflect the outcomes of non-surgical periodontal therapy. A continual loss of attachment three to six months post-therapy coupled with a reduction in biofilm lysine was suggested as an indication for adjunctive antibiotic therapy.

In addition, polyamines are involved in periodontal inflammation induced by endodontic infection. Polyamines have been detected in the root canals of teeth with necrotic pulps or periapical pathosis [50]. *Enterococcus*, *Pseudomonas*, *Lactobacillus*, and *Staphylococcus* are commonly found in infected root canals. They produce polyamines which induce the apoptosis of host leukocytes to cause cell death. Putrescine is a virulence factor with strong toxicity that contributes to the pain, foul odour, and periodontal destruction distinctive in anaerobic endodontic infections. Its levels are elevated in infected root canals with the spontaneous pain, soft tissue swelling, and tenderness on percussion characteristic of apical periodontitis [50,51]. Montis et al. reported statistically higher concentrations in the saliva of patients with chronic apical abscess. The authors suggested that the increase in salivary putrescine may be an adaptive response of bacteria in low pH environments to allow the alkalinisation of cellular cytosol and the production of a proton motive force effective for acid stress resistance and ATP production, thereby enhancing their viability [30].

### 3.3. Halitosis

Halitosis, or oral malodour, is defined as an unpleasant or offensive odour from the oral cavity and arises from tongue coatings, advanced carious lesions, and periodontal and peri-implant diseases [33,52]. It is a significant concern for the general population due to causing personal discomfort, social embarrassment, and compromised quality of life [34,53]. Halitosis is generated from putrefactive components of primarily gram-negative bacteria, but may also be derived from fungal, viral, and protozoal microorganisms [33]. These microbes produce volatile sulphur compounds such as hydrogen sulphide, methyl mercaptan, and dimethyl sulphide, which contribute greatly to halitosis. However, other odoriferous components such as indoles, skatoles, short-chain fatty acids, volatile organic acids, and polyamines play significant and at times dominant roles [54]. This review identified three studies of the effects of polyamines on halitosis [32,33,34]. Two studies utilised saliva samples and one collected oral biofilm on the tongue surface to evaluate its associations with polyamines.

Jo et al. identified two significantly increased polyamine metabolites, 5-aminovaleric acid and n-acetylornithine, in halitosis-affected saliva samples [33]. Cadaverine can be catabolized to 5-aminovaleric acid and is a putrefactive, pungent diamine associated with halitosis. This is consistent with findings by Zhang et al., which reported increased functional genes of cadaverine metabolic processes [32]. Jo et al. also reported a positive correlation between salivary cadaverine and halitosis. Salivary cadaverine and its metabolites, 5-aminovaleric acid and N-acetyl-cadaverine, are correlated with periodontitis, calculus, and plaque, which commonly contribute to malodour [33]. N-acetylornithine is converted to ornithine, the precursor of putrescine. Like cadaverine, putrescine is a foul-smelling polyamine associated with halitosis, plaque biofilm, and severe periodontitis [55]. Significant correlations of 5-aminovaleric acid and putrescine with *Alloprevotella* and 5-aminovaleric acid, n-acetylornithine, ornithine, and putrescine with *Prevotella* were also reported. Both are gram-negative, anaerobic bacteria commonly found in the halitosis-affected oral cavity, and findings suggest their close association with cadaverine and putrescine production [33].

Amines in biological fluids are often detected using high-performance liquid chromatography, capillary electrochromatography, and solid phase micro-extraction coupled with gas chromatography—mass spectrometry. The publication by Dadamio et al. was the only study in this review that incorporated in vivo analysis by means of a colorimetric chair side test to detect amines in saliva. Abundant levels of both putrescine and cadaverine were detected in saliva samples of the halitosis group, with higher levels of putrescine [34]. The substantial evidence of polyamines’ roles in halitosis may provoke novel therapeutic techniques, including potential ways to control, reduce, or modify their production. Further research is recommended to identify such methods for management.

## *4.* Discussion

This was the first publication that provided an overview of polyamines in dysbiotic oral conditions among older adults. The polyamine pathway involves a myriad of interactions between polyamines, their derivatives, related genes and mRNAs, precursors, metabolites, and enzymes (Figure 2). Dysregulation of these constituents signify dysbiosis in the oral cavity. The originality of this review lies in its focus on polyamines in dysbiotic oral conditions specifically among older adults, which is a relatively underexplored area in the literature. It contrasts with other literature that might focus on broader populations or on the systemic effects of polyamines, offering an important contribution by narrowing the focus to an aging demographic with unique oral health challenges. However, much of the data are drawn from existing studies, and new experimental findings are not provided.

The included studies of this review highlighted the potential use of polyamines as biomarkers for diagnosis and treatment monitoring. However, polyamines are involved in numerous physiological processes and hence can be altered in various conditions apart from oral diseases. Patients with dysbiotic oral conditions often present with other systemic diseases, which also affect polyamine levels. This lack of specificity may result in false positives. In addition, the complex involvement of multiple pathways and enzymes in polyamine metabolism may render the exact cause of dysregulation difficult to identify.

Several limitations are presented in this scoping review. Firstly, the literature search only included English publications from the year 2000 for standardization and to provide an exclusive review of the current literature. However, this may limit the search and exclude other potential, eligible articles published in earlier years or in other languages. Secondly, this study is a scoping review, which included publications conducted in diverse geographic locations but did not critically address differences in methodologies, populations, and diagnostic criteria. The quality of the studies were also not assessed. Thirdly, most of the cited references are correlational studies. Further research to identify cause-effect relationships are recommended to clarify the etiologiacal roles of polyamines in these dysbiotic oral conditions. Fourthly, most publications investigated on oral cancer and periodontal disease. Studies utilizing human samples are needed to collect further evidence of polyamines in other dysbiotic oral conditions among older adults, such as dental caries and oral candidiasis. Finally, only one publication focused purely on older adults, and thus, future studies specific to older aged groups are recommended to address the oral disease burden among the ageing population.

## 5. Conclusions

In conclusion, the current literature reported associations between polyamines and dysbiotic oral conditions among older adults. Dysregulated polyamine levels contribute to carcinogenesis, periodontal infection, and halitosis. Their involvement may warrant their use as biomarkers for early detection and the assessment of therapeutic outcomes. However, most of the included publications are laboratory studies, and clinical trials are essential to substantiate their clinical applications. Further understanding of disease-associated polyamine mechanisms is required to improve early diagnosis and the evaluation of treatment successes. If the associations of polyamines and dysbiotic oral conditions are confirmed, polyamines can be used as a simple and non-invasive tool to detect these conditions and monitor treatment progress in older adults.

## Figures and Tables

**Table 1:** Inclusion and Exclusion Criteria for Study Selection.

	Inclusion Criteria	Exclusion Criteria
**Study types**	Clinical studies and laboratory studies using human samples	Studies not on human samples, case reports, commentaries, letters and reviews
**Participants**	Included any participants aged ≥ 65	No participants aged ≥ 65
**Agents**	Reporting polyamines and related molecules	No reporting polyamines and related molecules
**Outcomes**	Reporting dysbiotic oral conditions	No reporting dysbiotic oral conditions

**Table 2:** Levels of Polyamines and Related Molecules in Dysbiotic Oral Conditions.

	Dysbiotic Oral Conditions		
	Oral Cancer	Periodontal Infection	Halitosis
**Polyamines**			
Cadaverine	**+**	**+**	**+**
Putrescine	**+**	**+**	**+**
Spermidine	**−**		
Spermine			
**Polyamine Derivatives**			
N-acetylputrescine	**+**		
N-acetylspermidine	**+**		
Butyrylputrescine	**+**	**+**	
N-acetylcadaverine	**+**	**+**	
N-acetylspermine	**+**		
**Polyamine-related genes**			
OAZ1	**+**	**+**	
SAT1	**+**	**+**	
ODC1	**+**		
SMOX	**+**		
SMS	**+**		
SRM	**+**		
Cadaverine-associated functional gene			**+**
**Polyamine Precursors**			
Arginine	**+**		
Lysine	**+**	**+/** **−**	
Ornithine	**+/** **−**	**+**	
**Metabolites**			
S-adenosylmethionine	**+**		
5-aminovaleric acid			**+**
N-acetylornithine			**+**

+ increased level; − decreased level; +/− conflicting results reported by different studies.

**Table 3:** Sources of Dysregulated Polyamines and Related Molecules in Dysbiotic Oral Conditions in Older Adults.

Source	Polyamine, or Related Molecule	Type of Study	Methodology	Potential Application	Country [Reference]
**Precancerous Conditions**					
Saliva	Putrescine, Cadaverine, Spermidine, Spermine	In vitro	CPSI-MS with ML	Detection	China [17]
Saliva	Polyamine related mRNA	In vitro	Sequence-specific primers and RT-qPCR	Detection	Greece [18]
Saliva	Putrescine	In vitro	CE-TOF-MS	Detection	Japan [19]
Saliva	Polyamine related mRNA	In vitro	Pre-amplification RT-qPCR with nested gene-specific primers	Detection	USA [20]
**Oral Cancer**					
Saliva, Cancerous tissue	Putrescine, Cadaverine, Spermidine	In vitro	CE-TOF-MS	Detection	Japan [21]
Saliva	Putrescine, Cadaverine	In vitro	CE-TOF-MS	Detection	USA [22]
Cancerous tissue	Putrescine, Spermidine	In vitro	LC-MS	Detection	China [23]
Saliva	Cadaverine	In vitro	CE-MS	Detection	Japan [24]
Saliva	Spermidine	In vitro	GC-MS	Detection	Brazil [25]
Saliva	Polyamine related mRNA	In vitro	Pre-amplification RT-qPCR with nested gene-specific primers	Detection	USA [26]
**Periodontal infection**					
Gingival crevicular fluid	Putrescine	In vitro	GC-MS	Detection	Brazil [27]
Gingival crevicular fluid	Putrescine	In vitro	GC-MS	Detection	Japan [28]
Gingival crevicular fluid	Putrescine, Cadaverine	In vitro	GC-MS, UH-LCTMS	Detection Monitor of therapy	USA [26]
Saliva	Putrescine, Cadaverine	In vitro	CE-TOF-MS	Detection	USA [22]
Saliva	Cadaverine	In vitro	UMS, UH-LCTMS	Detection	Germany [29]
Saliva	Polyamine related mRNA	In vitro	Pre-amplification RT-qPCR with nested gene-specific primers	Detection	USA [26]
Saliva	Putrescine	In vitro	GC-MS	Detection	Italy [30]
Biofilm from gingival crevice	Cadaverine	In vitro	LC	Detection Monitor of therapy	USA [31]
**Halitosis**					
Biofilm from tongue	Polyamine- associated functional gene	In vitro	LC-MS	Detection	China [32]
Saliva	Putrescine	In vitro	GC-MS	Detection	Korea [33]
Saliva	Putrescine, Cadaverine	In vitro	Chairside colorimetric test GC-MS	Detection	Belgium [34]

## Supplementary Materials

The following supporting information can be downloaded at: https://www.mdpi.com/article/10.3390/ijms251910596/s1.
