# Supplementary material for: Polyamines in Dysbiotic Oral Conditions of Older Adults: A Scoping Review
Source: Int J Mol Sci. 2024 Oct 1;25(19):10596. doi: 10.3390/ijms251910596 (PMC11477423; doi:10.3390/ijms251910596)
Supplement: Supplementary file 1 [file ijms-25-10596-s001.zip › ijms-3208985-supplementary.pdf]

Search Strategy

Time setting: 01/01/2000 onwards
Last search date: 28/05/2024

Scopus **744**

TITLE-ABS-KEY (polyamine) OR TITLE-ABS-KEY (polyamines) OR TITLE-ABS-KEY (putrescine) OR TITLE-ABS-KEY (spermidine) OR TITLE-ABS-KEY (spermine) OR TITLE-ABS-KEY (cadaverine) AND TITLE-ABS-KEY ("oral disease") OR TITLE-ABS-KEY ("oral diseases") OR TITLE-ABS-KEY ("mouth diseases") OR TITLE-ABS-KEY ("mouth disease") OR TITLE-ABS-KEY ("oral health") OR TITLE-ABS-KEY ("oral cavity") OR TITLE-ABS-KEY ("oral biomarkers") OR TITLE-ABS-KEY ("oral metabolites") OR TITLE-ABS-KEY ("oral microorganisms") OR TITLE-ABS-KEY (biofilm) OR TITLE-ABS-KEY ("oral biofilm") OR TITLE-ABS-KEY ("dental biofilm") OR TITLE-ABS-KEY (saliva) OR TITLE-ABS-KEY ("gingival crevicular fluid") OR TITLE-ABS-KEY (periodontal) OR TITLE-ABS-KEY (gingivitis) OR TITLE-ABS-KEY (periodontitis) OR TITLE-ABS-KEY ("periodontal disease") OR TITLE-ABS-KEY (periodontics) OR TITLE-ABS-KEY ("head and neck cancer") OR TITLE-ABS-KEY ("carcinoma, squamous cell") OR TITLE-ABS-KEY ("oral squamous cell carcinoma") OR TITLE-ABS-KEY ("squamous cell carcinoma of head and neck") OR TITLE-ABS-KEY ("mouth neoplasms") OR TITLE-ABS-KEY (leukoplakia) OR TITLE-ABS-KEY ("oral tumour") OR TITLE-ABS-KEY ("oral tumor") OR TITLE-ABS-KEY ("oral cancer") OR TITLE-ABS-KEY ("oral carcinoma") OR TITLE-ABS-KEY ("tooth disease") OR TITLE-ABS-KEY ("tooth diseases") OR TITLE-ABS-KEY ("dental caries") OR TITLE-ABS-KEY (xerostomia) OR TITLE-ABS-KEY (hyposalivation) OR TITLE-ABS-KEY (edentulism) OR TITLE-ABS-KEY (edentulous) OR TITLE-ABS-KEY (toothless) OR TITLE-ABS-KEY (denture) OR TITLE-ABS-KEY (candidiasis) OR TITLE-ABS-KEY (oral AND candidiasis) OR TITLE-ABS-KEY (oral AND thrush) OR TITLE-ABS-KEY (halitosis) OR TITLE-ABS-KEY (malodor) OR TITLE-ABS-KEY (malodour) OR TITLE-ABS-KEY ("bad breath") AND PUBYEAR > 2013 AND PUBYEAR < 2025

PubMed **1505**

(Polyamine OR "polyamines"[MeSH Terms] OR putrescine OR spermidine OR spermine OR cadaverine) AND (“oral disease” OR “oral diseases” OR "mouth diseases"[MeSH Terms] OR “mouth disease” OR “oral health” OR “oral cavity” OR “oral biomarkers” OR “oral metabolites” OR “oral microorganisms” OR biofilm OR “oral biofilm” OR “dental biofilm” OR saliva OR “gingival crevicular fluid” OR periodontal OR "gingivitis"[MeSH Terms] OR "periodontitis"[MeSH Terms] OR “periodontal disease” OR "periodontics"[MeSH Terms] OR “head and neck cancer” OR "carcinoma, squamous cell"[MeSH Terms] OR “oral squamous cell carcinoma” OR "squamous cell carcinoma of head and neck"[MeSH Terms] OR "mouth neoplasms"[MeSH Terms] OR “leukoplakia” OR “oral tumour” OR “oral tumor” OR “oral cancer” OR “oral carcinoma” OR “tooth disease” OR “tooth diseases” OR "dental caries"[MeSH Terms] OR dental caries OR xerostomia OR hyposalivation OR edentulism OR edentulous OR toothless OR denture OR candidiasis OR “oral candidiasis” OR “oral thrush” OR halitosis OR malodor OR malodour OR “bad breath”)

Web of Science **476**

(polyamine OR polyamines OR putrescine OR spermidine OR spermine OR cadaverine) (All Fields) and ("oral disease" OR "oral diseases" OR "mouth diseases" OR "mouth disease" OR "oral health" OR "oral cavity" OR "oral biomarkers" OR "oral metabolites" OR "oral microorganisms" OR biofilm OR "oral biofilm" OR "dental biofilm" OR saliva OR "gingival crevicular fluid" OR periodontal OR gingivitis OR periodontitis OR "periodontal disease" OR periodontics OR "head and neck cancer" OR "carcinoma, squamous cell" OR "oral squamous cell carcinoma" OR "squamous cell carcinoma of head and neck" OR "mouth neoplasms" OR "leukoplakia" OR "oral tumour" OR "oral tumor" OR "oral cancer" OR "oral carcinoma" OR "tooth disease" OR "tooth diseases" OR "dental caries" OR dental caries OR xerostomia OR hyposalivation OR edentulism OR edentulous OR toothless OR denture OR candidiasis OR "oral candidiasis" OR "oral thrush" OR halitosis OR malodor OR malodour OR "bad breath") (All Fields)
